# Hepatocarcinogenesis in Metabolic Dysfunction-Associated Steatotic Liver Disease (MASLD): Emerging Roles of Interleukin-10 and Transcriptomic Insights into IL-10 Signaling Rewiring

**DOI:** 10.3390/biomedicines14051093

**Published:** 2026-05-12

**Authors:** Helena Solleiro-Villavicencio, Lucía Angélica Méndez-García, Itzel Baltazar-Pérez, Pablo Fernando Pineda-Pérez, Ana Alfaro-Cruz

**Affiliations:** 1 Posgrado en Ciencias Genómicas, Universidad Autónoma de la Ciudad de México, Avenida San Lorenzo 290, Ciudad de México 03100, Mexico; 2 Licenciatura en Ciencias Genómicas, Universidad Autónoma de la Ciudad de México, Avenida San Lorenzo 290, Ciudad de México 03100, Mexico; 3 Laboratory of Immunometabolism, Research Direction, General Hospital of Mexico Hospital General de México “Dr. Eduardo Liceaga”, Ciudad de México 06720, Mexico; 4 Pathology Unit, General Hospital of Mexico Hospital General de México “Dr. Eduardo Liceaga”, Ciudad de México 06720, Mexico

**Keywords:** IL-10, MASLD, MASH, hepatocellular carcinoma, immunometabolism, IL-10 receptor, Scd2, Ddit4, hepatic microenvironment, transcriptomic reanalysis

## Abstract

Metabolic dysfunction-associated steatotic liver disease (MASLD) and its progressive inflammatory form, metabolic dysfunction-associated steatohepatitis (MASH), are increasingly recognized as key drivers of hepatocellular carcinoma (HCC). Unlike HCC caused by viral infections or alcohol, MASLD/MASH-related liver cancer develops within a chronic immunometabolic environment characterized by lipotoxicity, sterile inflammation, fibrogenesis, and remodeling of the microenvironment. In this setting, interleukin-10 (IL-10) has attracted growing attention due to its complex, context-dependent roles in immune regulation and tumor immune tolerance. This review explores IL-10 biology and its connection to MASLD/MASH-associated HCC, emphasizing the paradox that IL-10 may diminish harmful inflammation in early stages while promoting immunosuppressive conditions in advanced disease. To supplement existing research, we performed an exploratory reanalysis of publicly available bulk liver RNA-seq data from a mouse model that progresses from MASLD/MASH to HCC. The reanalysis revealed a receptor- and effector-specific rewiring of the IL-10 pathway: while the expression of canonical signaling genes (*Stat3*, *Jak1*, *Jak2*, *Tyk2*, *Socs3*) showed minimal changes across stages, receptor subunits (*Il10ra*, *Il10rb*) and IL-10-responsive effectors (such as *Scd2*, related to lipid metabolism, and *Ddit4*, involved in mTOR and glycolysis regulation) displayed strong stage-dependent induction. This was accompanied by a decrease in hepatocyte signature profiles and an increase in stromal and immune signatures. These results generate new hypotheses and raise key questions—particularly whether a large portion of IL-10 modulation originates from peripheral or non-parenchymal sources, and whether the transcriptional patterns observed reflect protein-level changes—that will require stage-specific, cell-focused human studies incorporating proteomic and cytokine measurements.

## 1. Epidemiological and Clinical Overview

Hepatocellular carcinoma (HCC) represents the most prevalent form of primary liver cancer, accounting for approximately 75–85% of primary hepatic tumors and ranking among the leading causes of cancer-related mortality worldwide [1]. The incidence of HCC varies geographically, reflecting regional differences in etiological factors and healthcare access.

Historically, most HCC cases were associated with the hepatitis B virus (HBV), hepatitis C virus (HCV), and alcohol-related cirrhosis. However, immunization programs, antiviral interventions, and public health policies have reduced the burden associated with the virus [2,3]. In contrast, increasing rates of obesity, insulin resistance, and metabolic syndrome have caused a significant increase in metabolic dysfunction-associated steatotic liver disease (MASLD, formerly NAFLD/MAFLD), now the leading cause of chronic liver disease globally, with a world prevalence of 32% in the adult population [2,3,4]. Recent studies indicate that 20–50% of HCC cases occur in people with MASLD, even in the absence of cirrhosis, and the annual incidence of MASLD-related HCC is expected to rise by 45–130% by 2030 [2,5,6]. These developments emphasize the rising significance of metabolic and inflammatory mechanisms in the development of liver cancer.

MASLD is defined by the accumulation of triglycerides in more than 5% of hepatocytes, while metabolic dysfunction-associated steatohepatitis (MASH) is further characterized by hepatocellular ballooning, lobular inflammation, and progressive fibrosis [7]. Approximately 20% of MASLD cases advance to MASH, and about 2% of these develop MASH-associated HCC. In contrast to viral or alcohol-induced HCC, MASH-related tumors frequently arise in non-cirrhotic livers, indicating distinct mechanisms of tumorigenesis and immune-metabolic regulation [8]. Recent studies highlight that the tumor microenvironment in MASH-associated HCC is marked by persistent, low-grade inflammation, altered lipid metabolism, and complex interactions between parenchymal and non-parenchymal hepatic cells [5,8].

The hepatic microenvironment consists of hepatocytes, cholangiocytes, liver sinusoidal endothelial cells (LSECs), hepatic stellate cells (HSCs), and a diverse array of immune cells, including Kupffer cells, dendritic cells (DCs), neutrophils, T and B lymphocytes, natural killer (NK) cells, and regulatory populations. Together, these cell types maintain hepatic homeostasis and regulate inflammatory responses [8]. In the context of MASLD and MASH, persistent metabolic stress and lipid accumulation foster an immunologically tolerant yet tumor-promoting environment. Among the principal immunomodulatory molecules involved, interleukin-10 (IL-10) is notable for its pleiotropic roles in sustaining hepatic homeostasis, modulating immune responses, and impacting tumor development [9]. The anti-inflammatory properties of IL-10 can promote immune tolerance, support the expansion of regulatory cell subsets, and potentially facilitate tumor progression. This dual functionality positions IL-10 as a critical mediator at the intersection of metabolic inflammation and hepatocarcinogenesis, justifying its detailed examination in subsequent sections.

## 2. Linking Metabolic Dysfunction, Inflammation, and Hepatocarcinogenesis

### 2.1. Mechanisms Mediating the MASLD-HCC Transition: Oxidative Stress, Lipotoxicity, and Chronic Inflammation

The progression from MASLD/MASH to HCC initiates with persistent metabolic injury that gradually compromises hepatocyte homeostasis. Toxic lipid species, such as saturated fatty acids (SFAs), lysophosphatidylcholine (LPC), and ceramides, activate NLRP3 inflammasomes and Toll-like receptor 4 (TLR4)-NF-κB signaling pathways in hepatocytes, LSECs, and Kupffer cells, thereby triggering inflammation and cellular stress [10]. In hepatocytes, SFAs also induce apoptosis via tumor necrosis factor-related apoptosis-inducing ligand receptor 2 (TRAIL-R2), caspase-8, FoxO3, and ER-stress-induced mitochondrial outer membrane permeabilization (MOMP), whereas in cholangiocytes, they activate caspases-3/7, also leading to apoptosis [10].

Palmitate further stimulates the c-Jun NH2-terminal kinase (JNK) pathway, which triggers the pro-apoptotic p53 upregulated modulator of apoptosis (PUMA)- Bcl-2-associated X protein (BAX) axis, as seen in hepatocyte lipoapoptosis models [11,12]. Additionally, JNK signaling is consistently elevated in MASH liver tissue [10]. Furthermore, pharmacological or genetic inhibition of JNK reduces free fatty acid (FFA)-mediated hepatocyte lipoapoptosis and fibrogenesis, underscoring the centrality of stress-kinase signaling in metabolic liver injury.

Lipotoxic hepatocyte death not only eliminates damaged cells but also propagates sterile inflammation by releasing damage-associated molecular patterns (DAMPs). It has been reported that ATP, high-mobility group box 1 (HMGB1), cholesterol crystals, mitochondrial DNA, and various extracellular RNAs (eRNAs) are released during lipotoxic apoptosis, necroptosis, or pyroptosis [13]. These DAMPs are sensed by pattern recognition receptors such as TLR3, TLR7, and TLR8 on Kupffer cells, infiltrating monocyte-derived macrophages, and even hepatocytes, leading to activation of NF-κB, TGF-β1, and the cellular communication network factor 2 (CCN2), thereby favoring fibrogenic progression [13].

Metabolic stress also leads to remodeling of the hepatic microvasculature. It has been reported that in high-fat and methionine-choline-deficient diet models, enlarged lipid-laden hepatocytes compress the sinusoidal lumina, particularly in the centrilobular regions, resulting in irregular sinusoidal contours, reduced blood flow, and altered Kupffer cell activity [14]. LSECs progressively lose their fenestrae, acquire a continuous basement membrane, and adopt a capillarized phenotype, diminishing oxygen diffusion and promoting local hypoxia, which, in turn, exacerbates lipotoxic injury, amplifies oxidative stress, and enhances angiogenic signaling. Experimental blockade of angiopoietin-2 halts HCC progression in MASLD models, highlighting that angiogenesis is not only a late feature of advanced tumor nodules but also an early step in carcinogenesis in steatotic, inflamed livers [14].

Epigenetic and transcriptional reprogramming further connect metabolic dysfunctions to the development of cancer. In that sense, Theys et al. (2024) demonstrated that loss of the peroxisome proliferator-activated receptor alpha (PPARα) function, either through hepatocyte-specific knockout or diet-induced DNA hypermethylation, triggers compensatory activation of lipid-sensing transcription factors and epigenetic writers and erasers [15]. This shift promotes a transition from pure metabolic stress to ferroptosis- and pyroptosis-associated lipotoxicity pathways, accompanied by increased oxidative damage and fibrogenesis. Complementary multi-omic analyses of human HCC revealed a metabolic profile characterized by increased de novo lipogenesis and heightened levels of cholesterol esters, phosphatidylcholines, sphingolipids, and triglycerides, all markedly altered in tumor tissue, showing the energetic and biosynthetic demands of malignant hepatocytes and the selective pressure imposed by chronic oxidative and lipotoxic stress [16].

Together, the evidence indicates that MASLD progression involves persistent lipotoxicity, stress-kinase activation, DAMP release, epigenetic remodeling, and microcirculatory disturbances. These factors, when combined, create a hepatic environment distinguished by high oxidative stress, ongoing cell death and regeneration, and an increased risk of genomic instability and malignant transformation.

### 2.2. Chronic Inflammation, Immune Dysregulation, and Establishment of a Tumor-Permissive Niche

In addition to the metabolic injury, MASLD and MASH are characterized by chronic inflammation that progressively reshapes the hepatic immune landscape. Early in the disease, activation of Kupffer cells by lipotoxic DAMPs and gut-derived signals leads to the secretion of cytokines and chemokines that recruit neutrophils and monocytes from the circulation [17]. Recruited monocytes differentiate into monocyte-derived macrophages, which often surround dying, fat-laden hepatocytes, forming so-called hepatic crown-like structures whose abundance correlates with disease severity [17]. A distinct subset of MASH-associated macrophages, defined by the markers triggering receptor expressed on myeloid cells 2 (TREM2)+, cluster of differentiation 9 (CD9)+, and glycoprotein non-metastatic melanoma protein B (GPNMB)+, accumulates in areas of cell death and extracellular matrix remodeling and produces osteopontin and galectin-3, thereby directly linking innate immune activation to fibrogenesis. Hepatocytes themselves contribute to this inflammatory environment by secreting hepatokines such as leukocyte cell-derived chemotaxin 2 (LECT2) and oncostatin M [18]. These factors promote inflammatory recruitment and HSC activation and have been implicated in angiogenesis and invasiveness, thereby further linking chronic steatohepatitis to carcinogenesis [19].

Adaptive immunity is likewise remodeled along the MASLD-HCC continuum. B cells are activated by gut dysbiosis and by antigens generated by oxidative stress, leading to chronic inflammation and fibrogenesis, in part by supporting monocyte-derived macrophage activity and immunoglobulin A (IgA)- Fc receptor γ (FcRγ) signaling [20]. CD8+ T cells and natural killer T (NKT) cells contribute to hepatocyte injury through cytotoxic mechanisms and by secreting tumor necrosis factor (TNF)-α and interferon (IFN)-γ, but chronic stimulation drives them toward exhaustion and dysfunction [21]. Metabolically activated, “auto-aggressive” CD8+ T cells capable of killing hepatocytes independently of classical major histocompatibility complex (MHC)-I recognition have been described in experimental MASH and illustrate how sustained metabolic stress can skew T-cell function toward tissue damage [17]. In parallel, exposure to excessive FFAs depletes CD4+ T cells, thereby weakening antitumor immune monitoring [21].

Cytokine signaling within this inflamed environment further promotes carcinogenesis. In the transition from MASH to HCC, IL-6 and TNF-α produced by inflammatory and stromal cells activate STAT3 in emerging tumor cells and upregulate CD44 and other survival signals, thereby overcoming p53-mediated growth arrest and apoptosis [21,22,23]. Additional molecular changes, including p62 accumulation and carnitine palmitoyltransferase-2 (CPT II) downregulation, protect HCC-initiating cells from oxidative stress and lipotoxicity, allowing them to succeed in the hostile metabolic microenvironment. MASLD-related HCC is enriched by mutations in genes such as *TERT* (telomerase reverse transcriptase), *CTNNB1* (catenin beta 1), *TP53* (tumor protein 53), and *ACVR2A* (activin A receptor type 2A); furthermore, cells also show a significant upregulation of de novo lipogenesis genes such as *SREBF1* (sterol regulatory element binding transcription factor 1), *FASN* (fatty acid synthase), *ACC* (acetyl-CoA carboxylase), *ACLY* (ATP citrate lyase) and *CD36*, further underscoring the tight coupling between metabolic reprogramming and oncogenic transformation [21,24,25].

Over time, this inflammatory setting evolves into an immunosuppressive tumor microenvironment. The neutrophils that are recruited through chemokines such as CXCL1, CXCL2, CXCL6, and CXCL8 generate ROS, secrete neutrophil elastase, form neutrophil extracellular traps (NETs), and activate HSCs [26]. On the other hand, NETs have been reported to influence CD4+ T cell differentiation into regulatory T cells (Tregs) by favoring a more anti-inflammatory transcriptomic program in the MASH liver microenvironment, where transforming growth factor beta (TGF-β), IL-10, and granulocyte colony-stimulating factor (G-CSF) are prominent [21,27]. Moreover, myeloid-derived suppressor cells expand, and T cells acquire an exhausted phenotype characterized by the expression of inhibitory receptors such as programmed death protein 1 (PD-1). This immunosuppressive milieu is characteristic of MASLD-related HCC, suggesting that immune checkpoint inhibitors may be less effective in this etiology than in viral HCC [28,29].

A critical mechanistic link between chronic inflammation and loss of antitumor immunity is provided by IL-10-producing IgA+ cells. In that sense, Shalapour et al. (2017) showed that in MASH, chronic inflammation and fibrosis were accompanied by the accumulation of liver-resident IgA+ plasma cells that expressed PD-L1 and IL-10 [30]. These cells directly suppress cytotoxic CD8+ T lymphocytes, which generally prevent the emergence of HCC.

Overall, metabolic dysfunction, lipotoxic stress, chronic inflammation, and immune remodeling work together to turn a steatotic and fibrotic liver into an environment that favors tumor development. In this process, IL-10 is a key immunoregulatory cytokine: it can reduce tissue damage and help resolve inflammation in the early stages of disease, but in advanced MASLD and early HCC, it contributes to the formation of a tolerogenic, immunosuppressive microenvironment. This dualistic, context-dependent function underpins the following sections, which focus on IL-10 signaling in hepatocarcinogenesis and its potential as a biomarker and therapeutic target in MASLD-related HCC.

## 3. Biology and Signaling of IL-10

IL-10 is a pleiotropic immunoregulatory cytokine that plays a central role in limiting and resolving inflammatory responses. It is produced by a wide range of cells, including monocytes and macrophages, dendritic cells, and regulatory and effector T cells, B cells, and several innate lymphoid populations. The importance of IL-10 for immune homeostasis is demonstrated in IL-10-deficient mice, which develop severe spontaneous colitis and systemic inflammation driven by uncontrolled responses to the commensal microbiota [31,32].

The general signaling architecture of IL-10 (receptor structure, JAK/STAT cascade, downstream metabolic and transcriptional outputs) is conserved across tissue contexts; the present section therefore draws on both hepatic and non-hepatic studies to establish this framework, while the subsequent Section 3.1 focuses specifically on hepatic IL-10 biology in liver homeostasis and chronic liver disease.

**Figure 1:**
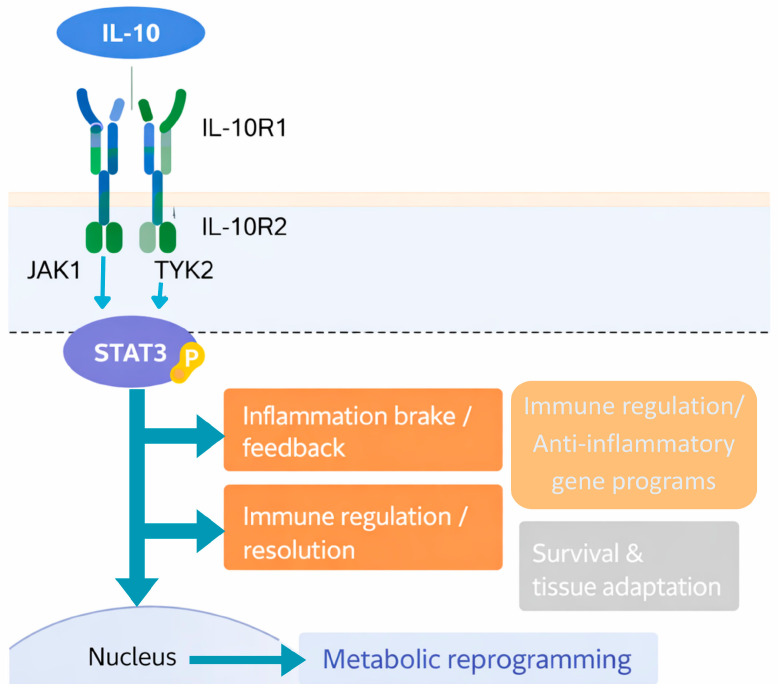
IL-10 signaling and downstream immunoregulatory and metabolic programs. Interleukin-10 (IL-10) signals through a heterotetrameric receptor complex composed of two IL-10R1 and two IL-10R2 chains, leading to activation of the associated kinases JAK1 and TYK2. Ligand engagement induces STAT3 phosphorylation, dimerization, and nuclear translocation, consequently promoting transcriptional programs that restrain inflammatory signaling and promote immune regulation. Downstream effects include feedback inhibition of pro-inflammatory pathways, resolution of immune responses, and induction of anti-inflammatory gene programs (e.g., *SOCS3*, *DDIT4*, and *SCD2*) that contribute to immune cell adaptation and metabolic remodeling under inflammatory stress. These effects are highly context-dependent and vary with cell type, timing, and the inflammatory milieu.

IL-10 signals through a heterotetrameric receptor complex composed of two ligand-binding IL-10 receptor 1 (IL-10R1/IL-10RA) chains and two accessory IL-10 receptor 2 (IL-10R2/IL-10RB) chains (Figure 1). IL-10R1 is expressed at higher levels on hematopoietic cells and confers ligand specificity, whereas IL-10R2 is more broadly expressed and mediates signaling for other IL-10 family cytokines [31,33]. Structural studies have detailed the architecture of the IL-10-IL-10R1 interaction and, more recently, the complete IL-10-IL-10R complex. These data show how receptor geometry and binding stoichiometry influence the strength and nature of downstream signaling, and they provide a foundation for designing IL-10 variants with targeted functional biases [34].

Ligand engagement of the IL-10 receptor induces conformational changes that bring the intracellular domains of IL-10R1 and IL-10R2 into proximity with their respective associated kinases, Janus kinase 1 (JAK1) and tyrosine kinase 2 (TYK2). Activated JAK1/TYK2 phosphorylate tyrosine residues in the receptor tails, creating docking sites for signal transducer and activator of transcription 3 (STAT3), which is the dominant mediator of IL-10 responses in myeloid cells [33,35]. STAT3 phosphorylation, dimerization, and nuclear translocation drive a transcriptional program that includes classical negative regulators of cytokine signaling, such as suppressor of cytokine signaling 3 (*SOCS3*) [36], metabolic stress adaptors like DNA damage-inducible transcript 4 (*DDIT4*), and enzymes such as the desaturase (*SCD2*) [32], collectively tuning inflammatory signaling, cell survival, and metabolic adaptation. Rather than globally shutting down myeloid cell activity, this STAT3-centered program selectively rewires transcriptional responses to pattern-recognition receptor stimulation, dampening pro-inflammatory gene expression while preserving other essential functions [35].

Functionally, IL-10 exerts multifaceted inhibitory effects on both innate and adaptive immunity. In antigen-presenting cells, IL-10 suppresses the production of pro-inflammatory cytokines such as TNF-α, IL-1β, IL-6, and IL-12; downregulates MHC-II and co-stimulatory molecules; and limits TLR-driven activation and maturation of DCs [31,33,35]. These actions together reduce the priming and effector function of Th1 and Th17 cells, restrain bystander tissue damage, and promote the resolution of inflammation. At the same time, IL-10 can preserve the viability of some immune subsets and, under certain circumstances, modulate or even enhance cytotoxic T cell responses, underscoring that its net impact is highly dependent on dose, timing, cellular source, tissue context, and the interactions with other cytokines and secreted molecules [31,34]. This dual aspect in IL-10 actions is exemplified by observations that IgA+ PD-L1+ IL-10-producing cells accumulate in inflamed livers and suppress liver-resident cytotoxic T cells that normally prevent HCC [30], whereas PEGylated IL-10, a long-acting IL-10 variant, and engineered IL-10 variants can expand and activate intratumoral CD8+ T cells and enhance responses to PD-1 blockade [31,37].

Recent work has added an important metabolic dimension to IL-10 biology. In macrophages, IL-10 signaling restrains inflammatory responses not only by altering gene expression but also by reshaping cellular metabolism. York et al. (2024) showed that IL-10 promotes the FFAs desaturation program and limits the accumulation of saturated very long-chain ceramides; in the absence of IL-10, TLR-activated macrophages accumulate these ceramides, which sustain an exacerbated inflammatory transcriptional profile through the transcription factor REL [32]. Moreover, IL-10-dependent induction of DDIT4 and SCD2 was identified as a critical axis controlling this mechanism, linking STAT3-driven transcriptional programs to mitochondrial function and lipid remodeling, thereby positioning lipid metabolism as an important downstream node through which IL-10 constrains pathological inflammation [32].

Taken together, these observations support the view of IL-10 as a central regulator of inflammatory tone, acting through tightly coordinated transcriptional and metabolic programs downstream of IL-10R-JAK1/TYK2-STAT3 signaling [31,32,33,34,35]. Its capacity to inhibit myeloid cell activation and shape T cell responses is important for preserving tissue stability, yet in chronic inflammatory settings, the same mechanisms can be co-opted to dampen anti-tumor immunity. In the liver, where IL-10 receptors are predominantly expressed on resident and infiltrating immune cells, this context-dependent activity is particularly relevant to the progression of MASLD and provides the conceptual basis for investigating additional functions of this cytokine in liver homeostasis, chronic hepatic injury, and hepatocarcinogenesis in the next sections.

### 3.1. IL-10 in Liver Homeostasis and Chronic Inflammatory Liver Disease

#### 3.1.1. IL-10 as a Regulator of Hepatic Immune Homeostasis

The liver is constantly exposed to dietary antigens, microbial products, and gut-derived metabolites, requiring tight immunoregulatory mechanisms for preventing exacerbated inflammation and tissue damage. IL-10 plays a central role in maintaining a tolerogenic environment by limiting inappropriate immune activation while preserving host defense. Early experimental and clinical observations established this cytokine as a key molecule restraining inflammatory responses in barrier organs, including the liver, by suppressing excessive proinflammatory mediators and immune-mediated injury [31].

Under physiological conditions, basal IL-10 signaling contributes to immune homeostasis by dampening responses to commensal-derived signals delivered via the portal circulation. Disruption of this regulatory axis results in uncontrolled inflammation, as evidenced by IL-10-deficient mice, which develop severe inflammatory disease driven by exaggerated responses to microbial cues [31,32]. These findings underscore the importance of IL-10 as a constitutive protective factor against immunopathology in metabolically and immunologically active organs, such as the liver.

#### 3.1.2. Cellular Sources of IL-10 During Liver Inflammation

During liver injury, IL-10 is produced by multiple immune populations, and its cellular sources vary with the nature and duration of the insult. Resident Kupffer cells and infiltrating monocyte-derived macrophages are major contributors to IL-10 production during acute and chronic hepatic inflammation, as established by mechanistic studies in human Kupffer cells in vitro [38] and in murine models of fulminant hepatitis [39]. These cells upregulate IL-10 in response to pattern recognition receptor stimulation (e.g., LPS/TLR signals), thus creating a negative feedback cycle that restricts immune activation [38,39].

Beyond macrophage populations, other immune subsets can also contribute to IL-10 production in inflamed liver tissue. DCs can adopt IL-10-associated tolerogenic programs under certain conditions, thereby limiting antigen-presenting cells (APC) maturation and co-stimulatory capacity and dampening downstream T-cell priming [33,35]. Likewise, Tregs and IL-10-producing effector-like regulatory subsets (often described as Tr1-like cells) can reinforce immune restraint in chronic inflammatory settings, thereby limiting collateral hepatocellular injury [40,41].

#### 3.1.3. IL-10 as a Regulator of Hepatic Inflammatory Tone Along with Antigen Presentation

A core function of IL-10 in inflamed tissues is to restrain excessive innate immune activation and dampen APC function. In myeloid cells, IL-10 reduces the production of proinflammatory cytokines and limits the expression of antigen presentation and co-stimulatory programs, thereby constraining amplification loops that sustain chronic inflammation and excessive T-cell priming [31,33,35]. Importantly, this immunoregulatory activity does not entail a global shutdown of immune function; rather, IL-10 imposes selective brakes on inflammatory gene expression and APC activation states, consequently reducing collateral tissue damage during persistent inflammatory stimulation [33,35].

In the liver, these effects are particularly relevant because antigen presentation is not restricted to classical professional APCs. LSECs can act as non-classical APCs. In vitro studies with rat primary liver sinusoidal endothelial cells co-cultured with T cells have shown that IL-10 can suppress T-cell activation induced by LSECs. This suppression occurs by decreasing antigen uptake through the mannose receptor and reducing the surface expression of accessory and co-stimulatory molecules such as MHC-II, CD80, and CD86 [42]. This observation supports the concept that IL-10 can modulate immune activation within the hepatic microenvironment at multiple levels, including by shaping antigen presentation and co-stimulatory signaling in resident hepatic cell types.

Collectively, these regulatory actions position IL-10 as a key determinant of hepatic inflammatory tone during injury, limiting cytokine-driven feed-forward loops and restraining antigen-presenting pathways that would otherwise perpetuate immune-mediated hepatocellular damage. At the same time, in chronic settings where injurious stimuli persist, sustained IL-10 signaling may result in a microenvironment defined by decreased effector activation and increased immune tolerance, with potential downstream consequences for disease progression, as explored in the following sections [31,34].

#### 3.1.4. IL-10 and Fibrogenesis: Modulation of Hepatic Stellate Cell Activation and ECM Remodeling

Progression from chronic liver injury to fibrosis is driven by sustained inflammatory signaling and HSC activation, leading to a myofibroblast-like phenotype and promoting extracellular matrix (ECM) deposition. In this setting, IL-10 has been studied primarily as an immunoregulatory mediator that can attenuate fibrogenic progression by reducing inflammatory cues that sustain HSC activation. Experimental studies in rat in vivo models of hepatic fibrosis [43], as well as complementary in vitro investigations with primary rat HSCs [44], and a more recent murine fibrosis model under thermoneutral conditions [45], have shown that IL-10 intervention influences the expression of matrix remodeling components in HSCs, such as matrix metalloproteinase-2 (MMP-2) and tissue inhibitor of metalloproteinase-1 (TIMP-1). This suggests an impact on ECM turnover rather than targeting a single profibrotic pathway [43]. 

Beyond ECM remodeling, additional studies suggest that IL-10 can indirectly influence profibrotic signaling by limiting proinflammatory cytokine production and immune-cell-driven amplification loops that promote HSC activation and collagen deposition, and by inducing HSC senescence [44,45]. However, the magnitude and durability of these effects appear to be context-dependent, and IL-10 alone is unlikely to reverse established fibrosis once a self-sustaining fibrogenic program has been consolidated. Thus, the most conservative interpretation is that IL-10 modulates the inflammatory–fibrogenic axis during chronic liver injury and may affect the rate of fibrogenesis but is insufficient as a single antifibrotic strategy.

#### 3.1.5. IL-10 in MASLD/MASH Inflammation: Compensatory Restraint and Context-Specific Trade-Offs

In MASLD/MASH, hepatic inflammation evolves in the setting of chronic metabolic stress and lipotoxic injury, during which sterile inflammatory signals and innate immune activation persist. Lipotoxic hepatocyte stress and death can release DAMPs, such as ATP, HMGB1, cholesterol crystals, mitochondrial DNA, and extracellular RNAs, which engage pattern-recognition receptors (PRRs) and amplify innate immune activation, thereby sustaining inflammation and promoting fibrogenic progression [13]. Within this environment, IL-10 is generally interpreted as part of a compensatory immunoregulatory response that limits excessive cytokine production and dampens antigen-presenting mechanisms, potentially restraining hepatocellular injury during early or active inflammatory phases [31,33,35]. However, because MASLD/MASH are conditions that are frequently prolonged, persistent IL-10 signaling may also result in a more tolerogenic immune tone by restraining effector activation, a state that can be protective early on yet may constrain immunological surveillance as disease progresses [31,34]. This context-dependent framework provides a mechanistic bridge to the following sections, which address how immunoregulatory circuits may intersect with the progressing tumor microenvironment in MASLD-associated HCC.

## 4. Experimental and Clinical Evidence Linking IL-10 to MASLD/MASH-Associated HCC

### 4.1. Mechanistic Anchors from Preclinical Studies

A strong mechanistic link between chronic steatohepatitis-associated inflammation and impaired anti-tumor immune surveillance was provided by Shalapour et al. by using MASLD/MASH-associated in vivo liver injury models (with supporting observations in human disease) [30]. The authors showed that chronic inflammation and fibrosis were accompanied by the accumulation of liver-resident IgA+ cells expressing PD-L1 and IL-10, which directly suppressed cytotoxic CD8+T lymphocytes that otherwise contribute to early tumor surveillance. Importantly, the study also delineated how the MASH liver microenvironment could support the generation of these IgA+ cells: class-switch recombination toward IgA was associated with a milieu enriched in factors known to promote IgA class switching, including TGF-β1, IL-21, and IL-33 [30]. In the livers of *MUP-uPA* mice fed a high-fat diet (HFD), IgA-B lineage cells were predominantly plasmablast/plasma cell-like and displayed high PD-L1 and IL-10 expression, whereas IgA-B lineage cells showed minimal PD-L1/IL-10 expression. Functionally, CD8+ T-cell ablation accelerated hepatocarcinogenesis; however, genetic or pharmacologic interference with IgA+ generation (disruption or blockade of key nodes in this axis, including PD-L1) attenuated HCC development and enabled cytotoxic T-cell-mediated regression of established tumors [30]. Collectively, these experiments support a causal framework in which inflammation-induced, IL-10-associated immunoregulatory circuits can dismantle CD8+-mediated surveillance and facilitate HCC outgrowth in the context of metabolic liver injury.

Recent cell-type-resolved analyses provide complementary mechanistic context for how such regulatory circuits may emerge during progression. In an in vivo single-cell transcriptomic study spanning MASH-to-HCC evolution, Huang et al. reported stage-associated remodeling of the hepatic immune microenvironment, with shifts in immune composition and functional states consistent with progressive reprogramming toward regulatory and dysfunctional/exhausted phenotypes [46]. While this work does not by itself establish IL-10 causality, it reinforces the larger concept that immune-cell state transitions accompany metabolic disease progression and that immunoregulatory programs can become increasingly prominent as malignancy develops, an important backdrop in the interpretation of bulk transcriptomic readouts, where downstream signaling nodes (e.g., STAT3-regulated pathways) may vary as inflammatory tone as well as cellular composition change, even when *Il10* transcript levels remain relatively stable at the tissue level.

### 4.2. Stage-Resolved Transcriptomic Evidence in a MASLD/MASH to HCC Mouse Model

To complement the mechanistic literature, we performed an exploratory reanalysis of publicly available bulk liver RNA-seq data from GEO (GSE246221), generated in a male streptozotocin (STZ)-treated HFD mouse model that recapitulates a stage-wise trajectory from metabolic liver injury through MASH and fibrosis to HCC [47]. Jeong et al. describe this model as progressively developing fatty liver, MASH, hepatic fibrosis, and HCC, with transcriptomic features that resemble those of metabolically driven human liver disease and MASLD-related HCC. To obtain a biologically homogeneous disease trajectory, we applied strict metadata curation: all pharmacological-intervention samples (Tirzepatide and matching vehicle controls, *n* = 10) and all HFD-only Batch 2 samples (*n* = 5) were excluded, yielding a final cohort of n = 40 biologically independent Batch 1 samples distributed across six stages (Table S1): Healthy control at 7 weeks (*n* = 5), Early MASLD at 14 weeks (*n* = 5), MASH at 20 weeks (*n* = 5), Liver fibrosis at 32 weeks (*n* = 5), Chronic non-tumor inflammation at 56 weeks (*n* = 6, representing STZ + HFD animals that did not progress to tumor formation), and HCC at 44–56 weeks (*n* = 14). Stage-specific histological grading of steatosis, lobular inflammation, and fibrosis is summarized in Table S1b. Because the analysis was performed on whole-liver tissue, expression patterns reflect the integrated hepatic microenvironment and cannot be unambiguously attributed to any specific cell type without single-cell or sorted-population validation [47]. A complete description of metadata curation, histological staging criteria (steatosis, lobular inflammation, and fibrosis grading as originally annotated by Jeong et al. [47] and aligned to the transcriptomic cohort in Table S1b), normalization (TMM plus log2-CPM), differential expression (limma-voom with robust + trend empirical Bayes [48], cross-validated with PyDESeq2 [49]), cell-type signature enrichment, statistical testing, and quality-control procedures is provided in Supplementary Materials.

**Figure 2:**
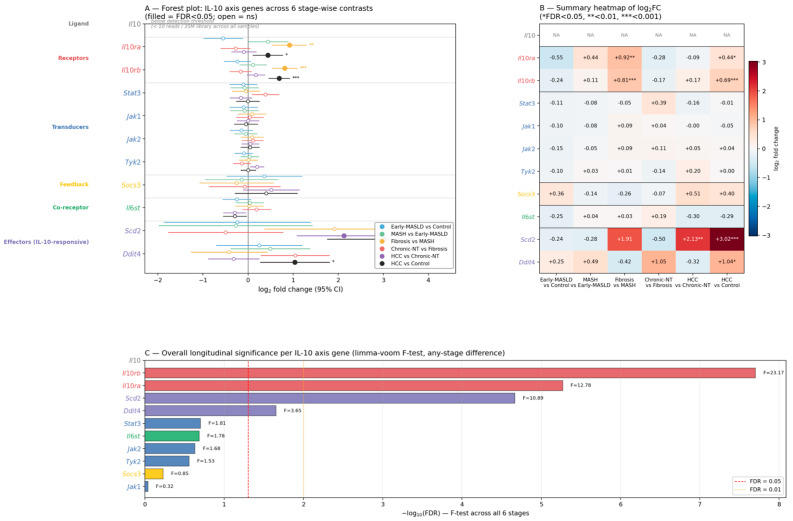
Stage-resolved relative dynamics of the IL-10 signaling axis in a MASLD/MASH-to-HCC mouse model. (**A**) Forest plot of log2 fold change (±95% confidence interval) for eleven IL-10 axis genes across six stage-wise contrasts in n = 40 samples (limma-voom with robust + trend empirical Bayes). Filled symbols indicate false discovery rate (FDR) < 0.05 (Benjamini–Hochberg); open symbols indicate non-significant contrasts. Genes are grouped into functional blocks (ligands, receptors, transducers, feedback, co-receptors, IL-10-responsive effectors). (**B**) Heatmap summarizing log2FC across contrasts, with significance stars (* FDR < 0.05, ** <0.01, *** <0.001). (**C**) Ranked bar plot of the longitudinal limma-voom F-test (any-stage difference) showing *Il10rb*, *Il10ra*, and *Scd2* as the most dynamic components of the axis. Data source: GSE246221 [47].

We examined the transcriptional behavior of eleven genes within the IL-10 signaling pathway, including the ligand (*Il10*), receptor subunits (*Il10ra*, *Il10rb*), the main transducer and Janus kinases (*Stat3*, *Jak1*, *Jak2*, *Tyk2*), the negative-feedback regulator *Socs3*, the *gp130* co-receptor (*Il6st*), and two recently identified IL-10-responsive effectors (*Scd2*, *Ddit4*) [32,50]. Throughout the six-stage comparisons, the axis did not function as a synchronized module (see Figure 2).

*Il10* expression was below the detection threshold (fewer than 10 reads per 35-million-read library across all 40 samples), making bulk RNA-seq quantification unreliable for this model. This should be interpreted with caution, as IL-10 mRNA is naturally low-abundance in whole-tissue samples, contains AU-rich elements that shorten its half-life, and is produced transiently by a small subset of cells. Dilution across tissue samples thus impairs its detectability [51,52]. Consistent with this, liver tissue from patients with MASLD shows IL-10 expression when measured by qPCR, ELISA [53], or immunohistochemistry. The sub-threshold detection of *Il10* in bulk RNA-seq reflects a sensitivity limitation of the assay for this tightly regulated, low-abundance cytokine transcript, rather than indicating its biological absence.

By contrast, the receptor subunits genes, *Il10ra* and *Il10rb*, exhibited pronounced stage-dependent upregulation, most notably in the Fibrosis *versus* MASH contrast (*Il10ra*, log2FC = +0.92, FDR = 2 × 10^−3^; *Il10rb*, log2FC = +0.81, false discovery rate (FDR) = 3 × 10^−4^) and in the HCC *versus* Control contrast (Il10ra, log2FC = +0.44, FDR = 0.037; Il10rb, log2FC = +0.69, FDR = 2 × 10^−5^). The longitudinal F-test identified *Il10rb* (F = 23.2, FDR < 10^−4^) and *Il10ra* (F = 12.8, FDR < 10^−4^) as the most dynamic components of the axis, followed by the IL-10-responsive effector *Scd2* (F = 10.9, FDR < 10^−4^), which showed particularly strong induction in HCC (HCC *versus* Control, log2FC = +3.02, FDR = 3.6 × 10^−4^; HCC *versus* Chronic-NT, log2FC = +2.13, FDR < 10^−2^). *Ddit4* also displayed stage-dependent induction (HCC *versus* Control, log2FC = +1.04, FDR = 0.03). In contrast, the transducers *Stat3*, *Jak1*, *Jak2*, and *Tyk2*, as well as the feedback regulator *Socs3* and the co-receptor *Il6st*, did not reach significance in the overall F-test after multiple testing correction (all FDR > 0.05). All differential expression results were replicated with PyDESeq2 (Wald test on rounded counts) as a sensitivity analysis, with log2FC values correlating strongly between methods across contrasts (Pearson r = 0.88–0.98; full results in Table S2a–c).

**Figure 3:**
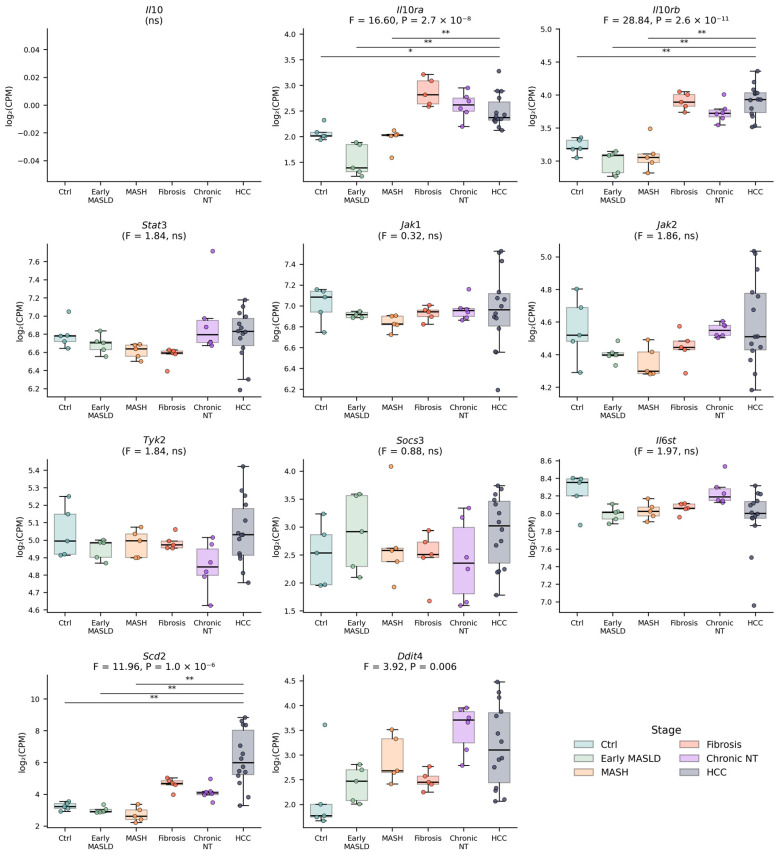
Stage-wise expression of IL-10 axis components in a MASLD/MASH-to-HCC mouse model. Box + swarm plots show normalized bulk liver RNA-seq expression values (log2-CPM) for the eleven IL-10 axis genes across the six disease stages (Control, 7 weeks, *n* = 5; Early MASLD, 14 weeks, *n* = 5; MASH, 20 weeks, *n* = 5; Liver fibrosis, 32 weeks, *n* = 5; Chronic non-tumor inflammation, 56 weeks, *n* = 6; HCC, 44–56 weeks, *n* = 14). For each gene, the overall one-way ANOVA F-statistic and *p*-value are shown; genes not reaching statistical significance are labeled “ns”. Horizontal brackets denote pairwise comparisons significant after Holm–Bonferroni correction (* FDR < 0.05, ** <0.01). *Il10* is shown using unfiltered log2 (CPM + 1) values because it did not pass the cohort expression filter and is therefore reported as sub-threshold. Data source: GSE246221 [47].

The absolute stage-wise expression of each axis component is shown in Figure 3. Consistent with the limma-voom F-test above, a one-way ANOVA on log2-CPM values (unweighted) confirmed the same genes as the stage-variable: *Il10ra* (F = 16.6, *p* = 2.7 × 10^−8^), *Il10rb* (F = 28.8, *p* = 2.6 × 10^−11^), and *Scd2* (F = 12.0, *p* = 1.0 × 10^−6^) showed highly significant stage-dependent variation, with pairwise Holm-adjusted Mann–Whitney comparisons confirming that Control, Early MASLD, and MASH differed significantly from HCC for each of these genes. *Ddit4* also varied across stages (F = 3.9, *p* = 6.5 × 10^−3^). No significant stage-dependent changes were observed at the transcript level for *Stat3*, *Jak1*, *Jak2*, *Tyk2*, *Socs3*, or *Il6st*. These results redirect the transcriptomic signal of the IL-10 axis in this model away from ligand abundance and classical transducer transcripts, and toward the surface receptor subunits and a specific subset of downstream effector genes described as IL-10-responsive in macrophages [32]. This is biologically plausible because cytokine signaling in tissues depends not only on ligand abundance but also on receptor availability, downstream signaling competence, and the wider cytokine milieu. An important methodological caveat concerns the stability of *Stat3* and *Socs3* mRNA: bulk RNA-seq measures transcript abundance, not pathway activity, and STAT3 activity is primarily regulated by post-translational tyrosine phosphorylation rather than by mRNA levels. STAT3 furthermore functions as a convergence point for multiple cytokine and growth factor pathways in the liver, including IL-6/gp130, IL-10, IL-22, leptin, and oncostatin M [54,55]; similarly, SOCS3 acts as a feedback regulator primarily within the IL-6/gp130/STAT3 axis [56] and is itself regulated post-transcriptionally. Accordingly, stable *Stat3* and *Socs3* transcript levels in bulk liver are compatible with substantial stage-dependent changes in STAT3 phosphorylation status and in the set of cytokine inputs converging on this node, which cannot be resolved by the present analysis and would require phospho-proteomic or phospho-flow approaches.

**Figure 4:**
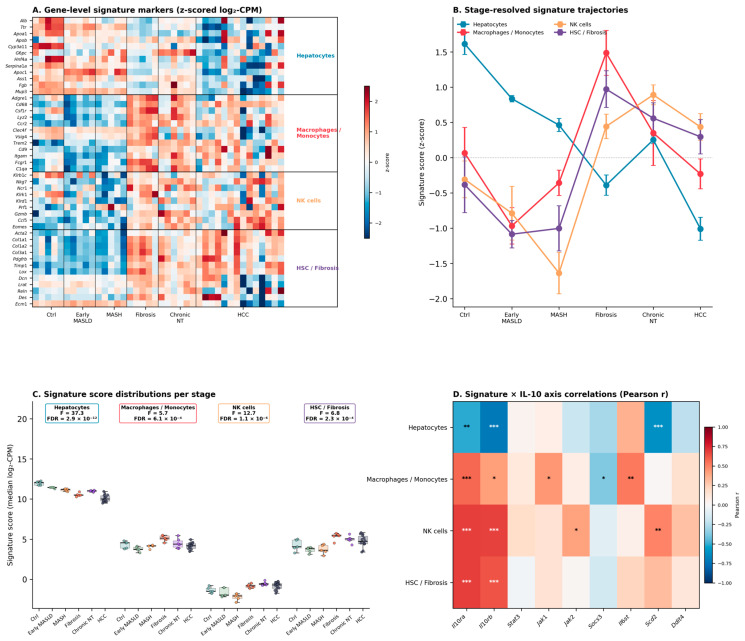
Cell-type gene signature profile remodeling across MASLD to HCC and its association with the IL-10 signaling axis. (**A**) Gene-level heatmap of the signature markers for four liver cells (hepatocytes, macrophages and monocytes, NK cells, hepatic stellate cells/fibrosis) across all 40 samples, ordered by stage (row-wise z-scored log2-CPM). (**B**) Stage-resolved trajectories (mean ± SEM, per- gene signature profile z-scored for display). (**C**) Per-signature distribution across stages (box + swarm plots) with one-way ANOVA F-statistics, BH-FDR, and Holm-adjusted pairwise comparisons (* FDR < 0.05, ** <0.01, *** <0.001). (**D**) Heatmap of Pearson correlations between gene signature profile scores and IL-10 axis gene expression across the 40 samples, with BH-FDR significance stars. Signatures were curated from CellMarker 2.0 [58] supplemented with mMCP-counter [59] and literature-canonical markers (Table S3). This is a signature-enrichment analysis; it is not a cell-proportion deconvolution. Data source: GSE246221 [47].

Because bulk RNA-seq conflates changes in per-cell expression with changes in cellular composition, we complemented the per-gene analysis with a cell-type signature enrichment approach focused on four liver cell gene signature profiles with complementary roles in the IL-10 axis: hepatocytes (parenchymal IL-10 targets expressing *Il10rb*), the macrophage/monocyte gene signature profile (including resident Kupffer cells, monocyte-derived macrophages, and *TREM2*^+^ lipid-associated macrophages, the principal IL-10 producers and targets in the liver [57], NK cells (innate lymphoid IL-10 sources in the NK-rich liver), and hepatic stellate cells (HSC/fibrosis effectors and anti-fibrotic IL-10 targets). This is a signature-enrichment approach, not a deconvolution: it reports relative enrichment of gene-set scores across stages rather than absolute cellular proportions. Gene signatures were curated as a consensus panel from CellMarker 2.0 [58] filtered to liver-tissue entries, supplemented with literature-canonical markers for disease-relevant subsets, and scored per sample as the median log2-CPM of the detected signature genes, following the rationale of mMCP-counter [59] (full signature panel and gene-level provenance in Table S3; per-sample scores in Table S4a). The four gene signature profiles showed pronounced and coherent stage-dependent remodeling (Figure 4A–C and Table S4b): the hepatocyte signature declined progressively from Control to HCC (F = 37.3, FDR < 10^−4^); the HSC/Fibrosis signature peaked at the Liver fibrosis stage (32 weeks) and remained elevated in Chronic non-tumor inflammation and HCC (F = 6.8, FDR = 2 × 10^−4^); the NK cell signature increased from MASH onward (F = 12.7, FDR < 10^−4^); and the macrophage/monocyte signature expanded progressively from Fibrosis onward (F = 5.7, FDR = 6 × 10^−4^). Together, these dynamics are consistent with a transition of the tissue transcriptome from a parenchymal-dominated profile in health to a profile enriched in stromal and immune gene signatures in advanced disease.

Taken together, these data support a conservative interpretation: bulk *Il10* mRNA abundance alone is insufficient to infer the IL-10 pathway state during MASLD/MASH-associated hepatocarcinogenesis. Rather than a synchronously regulated module, the IL-10 axis in this model shows a receptor- and effector-centered transcriptomic signature—dominated by *Il10ra*, *Il10rb*, *Scd2*, and *Ddit4*—that tracks the progressive contraction of the parenchymal hepatocyte gene signature profiles and the concomitant expansion of stromal (HSC) and immune (macrophage/monocyte and NK) gene signature profiles during the MASLD to HCC transition. A key implication of this receptor/effector-dominated signature, combined with the sub-threshold detection of *Il10* mRNA in bulk tissue, is that the ligand driving the observed pathway activity need not be produced locally in the hepatic parenchyma at any given time [53]; a substantial fraction may plausibly originate from peripheral or non-parenchymal sources, including IL-10-producing plasma cells, regulatory B cells, and systemic IL-10 pools documented in obesity and metabolic inflammation, or from transient infiltrates of IL-10-producing cells whose mRNA signal is diluted in bulk measurements.

Importantly, this rewiring is likely to be specific to the metabolic context of MASLD/MASH rather than a generic feature of chronic liver injury. Hepatic inflammation driven by lipotoxicity differs fundamentally from that driven by viral or alcoholic insults in the nature of its signaling substrates and in the cellular composition of the resulting myeloid gene signature profile. Beyond classical M1/M2 polarization of resident cells, MASLD is characterized by a genuine turnover of the macrophage pool: embryo-derived resident Kupffer cells, which in the healthy liver express a lipid-handling, metabolically specialized rather than prototypically pro-inflammatory transcriptional program, are progressively lost during MASH and replaced by bone-marrow-derived monocytes that repopulate the Kupffer niche (monocyte-derived Kupffer cells) and additionally give rise to the MASH-associated TREM2^+^/Osteopontin^+^ lipid-associated macrophage (LAM) gene signature profile, which localizes to regions of hepatocyte damage, fibrotic septa, and reduced resident-Kupffer cell density [57]. This resident-to-recruited macrophage transition is not conserved across etiologies; it is particularly prominent in diet-induced MASLD/MASH, less pronounced in most viral hepatitis contexts, and therefore shapes cytokine output—including the IL-10 axis—in a lipotoxicity-specific manner. In parallel, lipotoxic DAMPs and bioactive lipid species such as SFAs, ceramides, and sphingosine directly reprogram macrophage bioenergetics toward glycolysis and alter their cytokine profiles (Section 2.1), and the IL-10-responsive effector Scd2—identified in macrophages as a central node linking IL-10 signaling to sphingolipid metabolism and inflammation control [32]— is itself embedded in lipid-metabolic remodeling and is therefore expected to behave differently in a lipotoxic microenvironment than in a viral or autoimmune one. Against this background, the stage-specific induction of *Il10ra*, *Il10rb*, and *Scd2* observed in the present model, together with the coordinated expansion of the macrophage/monocyte signature across Fibrosis, Chronic-NT, and HCC stages (Figure 4), can be read as an integrated readout of a lipotoxic microenvironment that simultaneously remodels the identity, origin, and metabolic state of the hepatic macrophage pool and, as a consequence, the IL-10 receptor-effector circuit—rather than as a canonical ligand-driven feedback response.

Addressing these limitations will require a convergent multi-modal strategy. Single-cell and single-nucleus RNA-sequencing of the MASLD-to-HCC continuum would allow direct attribution of *Il10*, *Il10ra*, *Il10rb*, *Scd2*, and *Ddit4* expression to specific hepatic cell types and subsets, resolve the TREM2^+^ LAM gene signature profile and its IL-10-producing subfraction, and discriminate between cell-intrinsic rewiring and compositional shifts. Assay for transposase-accessible chromatin sequencing (ATAC-seq) could complement this view by identifying whether the induction of *Il10ra* and *Il10rb* in advanced stages is accompanied by chromatin-level priming of regulatory elements, and by revealing stage- and cell-type-specific transcription-factor footprints compatible with receptor-biased rewiring. Spatial transcriptomics would enable the localization of IL-10 axis activity relative to histological landmarks such as fibrotic septa, zonation gradients, immune aggregates, and tumor margins, which is particularly relevant in a disease that evolves in a spatially heterogeneous manner. Phospho-proteomic and phospho-flow approaches would directly test the hypothesis that STAT3 activity, rather than its mRNA abundance, conveys the stage-dependent signal of IL-10 pathway engagement and would additionally dissect the contributions of convergent cytokine inputs (IL-6, IL-22, leptin, oncostatin M) that share STAT3 as a transducer. Finally, targeted measurement of IL-10 protein in liver tissue and in portal and systemic plasma, by ELISA, multiplexed immunoassay, or tissue proteomics, would directly test the extrahepatic-origin hypothesis proposed above and clarify whether local hepatic IL-10 is below the detection limit of bulk mRNA profiling yet detectable and functionally relevant at the protein level.

**Figure 5:**
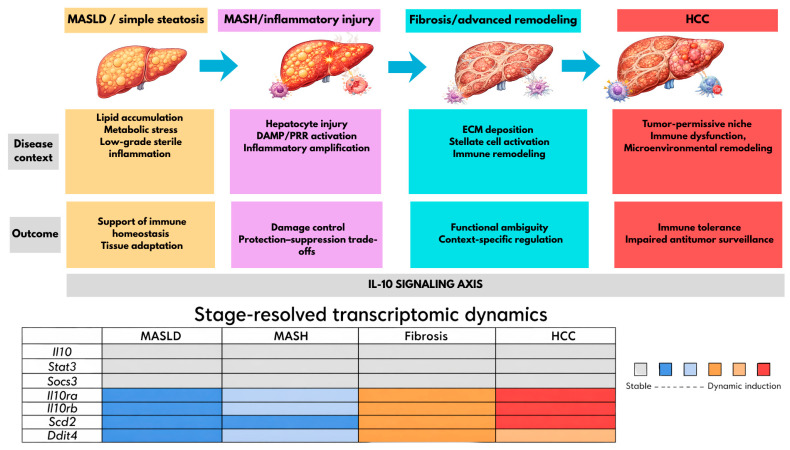
Conceptual model of IL-10 axis rewiring across the MASLD–MASH–fibrosis–HCC continuum. Top panels depict histopathological progression (lipid accumulation in MASLD; hepatocyte injury and DAMP activation in MASH; ECM deposition and stellate cell activation in fibrosis; tumor-permissive niche in HCC) and corresponding IL-10 outcomes (from support for immune homeostasis, through damage control with protection–suppression trade-offs and context-specific regulation in fibrosis, to immune tolerance and impaired antitumor surveillance in HCC). The bottom heatmap (Stage-resolved transcriptomic dynamics) summarizes the bulk RNA-seq reanalysis of GSE246221: the IL-10 ligand (*Il10*) and canonical transducers (*Stat3*, *Socs3*) remain stable across stages, whereas receptor subunits (*Il10ra*, *Il10rb*) and IL-10-responsive effectors (*Scd2*, *Ddit4*) show progressive, stage-dependent induction. This receptor- and effector-centered pattern supports the hypothesis that modest changes in ligand abundance may yield substantial biological effects by reconfiguring the IL-10 signaling infrastructure rather than by ligand amplitude alone (Section 4.3). Color gradient: gray = stable; blue → red = increasing dynamic induction.

Independent human confirmation of the framework proposed here is addressed in Section 4.3, and broader priorities for the field, including the need for well-annotated retrospective human cohorts across the MASLD/MASH-to-HCC continuum [60], are discussed in Section 5. Within the scope of the present reanalysis, the specific hypotheses that follow from Figure 2, Figure 3 and Figure 4 and that remain to be tested are: (i) the receptor- and effector-focused transcriptomic IL-10 axis signatures (*Il10ra*, *Il10rb*, *Scd2*, *Ddit4*) are preserved in human MASLD-associated HCC; (ii) that the negative correlation between the hepatocyte signature and IL-10 receptor expression reflects a genuine compositional shift at the single-cell level rather than a purely transcriptional rewiring within a fixed cell population; and (iii) that a measurable fraction of the IL-10 ligand modulating hepatic receptor-expressing cells in MASLD originates from non-parenchymal or extrahepatic sources. Until such validation is available, the present reanalysis positions the IL-10 axis in MASLD-associated hepatocarcinogenesis not as a unidirectional anti- or pro-tumorigenic pathway, but as a compositionally and metabolically remodeled receptor-effector circuit whose net effect is determined by the specific cellular and lipid-microenvironmental context in which it operates (Figure 5). Although some aspects of the IL-10R/STAT3/SOCS3 signaling pathway have been linked to MASLD and MASH-related liver cancer development.

### 4.3. Human and Translational Considerations

Although direct human evidence linking IL-10 to MASLD/MASH-associated hepatocarcinogenesis remains limited, available studies support the relevance of IL-10-associated immunoregulatory programs in metabolic liver disease. More recent preclinical work by Petriv et al. (2024) characterized regulatory B cell (Breg) subsets highly expressing PD-L1 and IL-10 in the livers of mice with MASLD and HCC, with analogous populations verified in smaller human cohorts of HCC tissue and MASLD blood samples [61]. Moreover, human biopsy-based data further indicate that hepatic and circulating IL-10 levels decrease modestly yet significantly as lobular inflammation worsens in patients with obesity [53], suggesting that even subtle variations in ligand abundance may track with meaningful shifts in hepatic inflammatory status. Together, these studies establish that IL-10 biology in human MASLD is dynamic and context-specific, varying across disease stages. However, they do not address the mechanistic question of how the IL-10 signaling axis itself is modulated—whether through receptor availability, effector induction, or ligand source. Our stage-resolved transcriptomic reanalysis begins to address this gap by revealing that, in a MASLD/MASH-to-HCC mouse model, stable bulk ligand expression coexists with robust stage-dependent induction of the receptor subunits (*Il10ra*, *Il10rb*) and downstream effectors (*Scd2*, *Ddit4*). This observation raises the possibility that modest changes in IL-10 ligand levels—as reported in human biopsies—may nevertheless translate into substantial biological effects by reconfiguring the receptor-effector infrastructure rather than by ligand amplitude alone. The investigation of whether this receptor- and effector-centered reorganization is maintained in human MASLD-associated HCC, and whether the modulating ligand predominantly originates from non-parenchymal or extrahepatic sources, thereby presents a specific, hypothesis-driven priority for future studies utilizing cell resolution in human subjects. Although direct evidence linking specific peripheral IL-10 sources to the hepatic IL-10 pool in MASLD-HCC is currently lacking, plausible candidate compartments include the visceral adipose tissue, where adipose-resident regulatory T cells, M2-like macrophages and B cells produce IL-10 in the broader context of metabolic inflammation [62], and the gut–liver axis, which has been mechanistically linked to hepatic IL-10 output: in murine models of intestinal barrier disruption, microbial signals reaching the liver via portal circulation directly induce an IL-10-producing hepatic macrophage phenotype [63]. Whether IL-10 originating from these compartments contributes meaningfully to the hepatic axis output during MASLD-HCC progression remains, to our knowledge, an open question that requires direct measurement.

An important limitation of current human research is the lack of a comprehensive, stage-specific, etiology-specific public dataset spanning the full MASLD/MASH-to-HCC progression. Existing cohorts are largely fragmented by disease stage, and even resources such as GEO GSE164760, which includes human MASH and MASH-HCC samples, are cross-sectional and rely on microarray data. This restricts the ability to track within-subject trajectory inference and achieve detailed transcript-level insights and specifically precludes testing the receptor- and effector-centered hypotheses proposed above: the induction dynamics of *IL10RA*, *IL10RB*, *SCD2*, and *DDIT4* across MASLD, MASH, fibrosis, and HCC stages cannot be resolved without a stage-spanning longitudinal human cohort with single-gene resolution. Additionally, although some aspects of the IL-10R-mediated signaling pathway have been linked to MASLD and MASH-related liver cancer development, fully understanding its role in humans remains limited. These gaps demonstrate the need for well-characterized retrospective cohorts that include defined stages of MASLD/MASH-associated hepatocarcinogenesis, ideally with RNA-seq data and, when possible, with cell-resolved or spatial transcriptomic approaches [55,64].

## 5. Conclusions and Future Directions

MASLD/MASH-associated hepatocarcinogenesis develops within a chronic immunometabolic landscape formed by lipotoxic injury, sterile inflammation, fibrogenesis, and progressive remodeling of the hepatic microenvironment. Within this continuum, IL-10 emerges not as a uniformly protective or homogeneous tumor-promoting cytokine, but as a context-dependent regulator whose biological significance varies with disease stage, cellular source, and tissue organization. In early disease, IL-10 may help restrain excessive inflammatory damage and preserve hepatic immune homeostasis; however, in more advanced settings, persistent or context-specific IL-10-associated programs may also contribute to immune tolerance and impaired antitumor surveillance.

By integrating mechanistic and translational evidence with stage-specific transcriptomic reanalysis of a MASLD/MASH-to-HCC murine model, this review advocates for a receptor- and effector-focused interpretation of the IL-10 axis in hepatocarcinogenesis. Our analysis indicates that this pathway does not operate as a synchronously regulated module throughout disease progression. Instead, the IL-10 ligand and the canonical transducers (*Stat3*, *Socs3*) remain transcriptionally stable across different stages, whereas the receptor subunits (*Il10ra*, *Il10rb*) and IL-10-responsive effectors (*Scd2*, *Ddit4*) demonstrate strong stage-dependent induction. This pattern implies that the functional output of the axis in MASLD-HCC is unlikely to be fully captured by ligand abundance alone, thereby supporting the hypothesis that modest alterations in ligand levels may lead to significant biological effects through the reconfiguration of receptor-effector architecture rather than through ligand amplification.

Significant gaps in knowledge still exist. Human data remain incomplete, as there is no publicly available, stage-specific, etiology-focused dataset covering the entire MASLD/MASH-to-HCC progression. Future research should focus on well-annotated retrospective human cohorts that include clearly defined stages of MASLD/MASH-related HCC, ideally with RNA sequencing and, where possible, single-cell, cell-sorted, or spatial analyses. These approaches will be crucial to identify which cell types produce IL-10, which express IL-10 receptor components, and how pathway responses change during disease progression. A testable hypothesis from this review is whether a large part of the IL-10-related hepatic signaling in MASLD-HCC stems from peripheral or non-parenchymal sources, which would need additional proteomic and cytokine measurements in portal and systemic plasma.

Although the IL-10 axis may ultimately have therapeutic relevance in MASLD/MASH-associated HCC, current evidence does not support specific therapeutic claims. The pleiotropic nature of IL-10, the pathway rewiring observed during disease progression, and the still-incomplete resolution of this circuitry in human disease together imply that any future therapeutic strategy targeting this axis cannot be pursued as a stage-independent intervention. Validating the receptor- and effector-centered hypotheses proposed in this review will be a necessary step before any rational therapeutic evaluation can be undertaken.

## Data Availability

The custom bioinformatic pipeline and processed datasets generated in this study are openly available in GitHub at https://github.com/Hsolleiro/GSE246221_IL10_Analysis. These data were derived from the following public-domain resource: the NCBI Gene Expression Omnibus (GEO), accession number GSE246221 (https://www.ncbi.nlm.nih.gov/geo/query/acc.cgi?acc=GSE246221). accessed on 19 April 2026.

## Supplementary Materials

The following supporting information can be downloaded at: https://www.mdpi.com/article/10.3390/biomedicines14051093/s1, Supplementary Methods: Bioinformatic re-analysis of GSE246221. Supplementary Tables (Excel file): Table S1a. Cohort; Table S1b. Histology; Table S2a. Limma Contrasts Top 50; Table S2b. F-test Top 200; Table S2c. DESeq2 HCC vs. Control; Table S3. Signature Panel; Table S4a. Sample Scores; Table S4b. Signature ANOVA. Supplementary Figure S1. Quality Control of the Curated Cohort.

## Abbreviations

ANOVA, analysis of variance; APC, antigen-presenting cell; BMDM, bone marrow-derived macrophage; Breg, regulatory B cell; CPM, counts per million; DAMP, damage-associated molecular pattern; DC, dendritic cell; DDIT4, DNA damage-inducible transcript 4; DEG, differentially expressed gene; ECM, extracellular matrix; FDR, false discovery rate; GEO, Gene Expression Omnibus; HCC, hepatocellular carcinoma; HFD, high-fat diet; HSC, hepatic stellate cell; IgA, immunoglobulin A; IL, interleukin; IL-10R, interleukin-10 receptor; IL10RA/IL10RB, interleukin-10 receptor alpha/beta subunits; JAK, Janus kinase; KC, Kupffer cell; LAM, lipid-associated macrophage; log2-CPM, log2 counts per million; LSEC, liver sinusoidal endothelial cell; MASLD, metabolic dysfunction-associated steatotic liver disease; MASH, metabolic dysfunction-associated steatohepatitis; MDSC, myeloid-derived suppressor cell; MoMF, monocyte-derived macrophage; NAFLD, nonalcoholic fatty liver disease; NASH, nonalcoholic steatohepatitis; PCA, principal component analysis; PD-L1, programmed death-ligand 1; PRR, pattern-recognition receptor; RNA-seq, RNA sequencing; SCD2, stearoyl-CoA desaturase 2; SOCS3, suppressor of cytokine signaling 3; STAT3, signal transducer and activator of transcription 3; STZ, streptozotocin; TGF-β, transforming growth factor beta; TLR, Toll-like receptor; TMM, trimmed mean of M-values; Tr1, type 1 regulatory T cell; Treg, regulatory T cell; TREM2, triggering receptor expressed on myeloid cells 2; TYK2, tyrosine kinase 2.
