# Supplementary material for: Hepatocarcinogenesis in Metabolic Dysfunction-Associated Steatotic Liver Disease (MASLD): Emerging Roles of Interleukin-10 and Transcriptomic Insights into IL-10 Signaling Rewiring
Source: Biomedicines. 2026 May 12;14(5):1093. doi: 10.3390/biomedicines14051093 (PMC13204704; doi:10.3390/biomedicines14051093)
Supplement: Supplementary file 1 [file biomedicines-14-01093-s001.zip › biomedicines-4237763-supplementary-resubmit_formatted.pdf]

biomedicines

MDPI

Supplementary Methods: Bioinformatic re-analysis of GSE246221

This Supplementary Data section describes the complete bioinformatics pipeline used to reanalyze the publicly available STZ+HFD mouse liver RNA-seq dataset GSE246221 and to generate Figures 2–4 of the main manuscript. All results are produced by a custom Python pipeline that is openly available at https://github.com/Hsolleiro/GSE246221_IL10_Analysis

S1 Data source and study design

Publicly accessible bulk liver RNA-seq data were obtained from the NCBI Gene Expression Omnibus (GEO) under accession GSE246221 (Jeong et al., 2024). The dataset was generated from a male mouse model induced with streptozotocin (STZ) and a high-fat diet (HFD), which recapitulates the metabolic and fibrotic progression of MASLD/MASH to hepatocellular carcinoma (HCC). For the present re-analysis, a well-defined subset was selected to examine the natural history of disease without confounding experimental factors (see § S2). The analysis focuses on stage-specific changes, potential decoupling, and transcriptional rewiring of the IL-10 signaling axis during hepatocarcinogenesis.

S2 Metadata curation and stage assignment

Sample metadata were curated from the GEO series matrix and the associated SRA run table. To ensure a clean biological trajectory of disease progression and minimize technical confounders, the following samples were strictly excluded from the re-analysis:

- Samples receiving pharmacological interventions (Tirzepatide and matching vehicle controls; *n*=10), to avoid drug-induced transcriptomic effects unrelated to natural disease progression.
- HFD-only samples from sequencing Batch 2 (*n*=5; Batch2_HFDonly20w_1–5), which differ from the main STZ+HFD trajectory in both intervention (no STZ) and sequencing batch. This exclusion prevents confounding of biological stage effects with technical batch effects.
- Samples with ambiguous histological annotations were not retained.

The final curated cohort comprises *n*=40 biologically independent samples, all from sequencing Batch 1, male, wild-type C57BL/6J, classified into six stages that follow age, diet, STZ treatment, and histological annotation (Table S1):

- Stage 1 — Healthy control: 7 weeks, standard chow (*n*=5)
- Stage 2a — Early MASLD: 14 weeks, STZ+HFD (*n*=5)
- Stage 3 — MASH (peak inflammation): 20 weeks, STZ+HFD (*n*=5)
- Stage 4 — Liver fibrosis: 32 weeks, STZ+HFD (*n*=5)
- Stage 2b — Chronic inflammation background: 56 weeks, non-tumor adjacent tissue (*n*=6)
- Stage 5 — HCC (tumor): 44–56 weeks, confirmed neoplastic lesions (*n*=14)

Stage 2b (Chronic inflammation / non-tumor) represents hepatic tissue from STZ+HFD animals at 56 weeks that did not progress to tumor formation and therefore serves as a longitudinal background control for the HCC cohort (Stage 5), rather than as a step in the canonical MASLD→MASH→Fibrosis→HCC sequence. The full distribution of histological grades for each stage is provided in Table S1b.

S3 Processing of raw expression data

Raw RSEM-quantified transcript counts were obtained from the GEO submission and processed using a custom Python pipeline (Python v3.12) built on pandas, NumPy, SciPy, statsmodels, scikit-learn, matplotlib, and seaborn. Gene identifiers were harmonized to official MGI mouse gene symbols by retaining the "ENSMUSG_Symbol" composite identifier throughout the pipeline. When multiple transcripts mapped to the same gene symbol, the RSEM-estimated counts were summed at the gene level prior to normalization.

Because RSEM estimates can be fractional, counts were retained as floating-point values for the primary differential expression analysis (limma-voom, §S5.1) and rounded to non-negative integers only for the PyDESeq2 sensitivity analysis (§S5.2).

Low-expression filtering was applied at the cohort level: genes were retained only if a CPM ≥ 0.5 was observed in at least 3 samples, yielding 20,100 genes available for statistical analysis. The IL-10 ligand gene *Il10* did not pass this filter (< 10 reads per 35M-read library across all 40 samples) and is explicitly marked as sub-threshold throughout the analysis.

S4 Normalization

TMM (Trimmed Mean of M-values) normalization factors were computed across all 40 samples, following the formulation of Robinson & Oshlack (2010). Effective library sizes were obtained by multiplying the raw library size by the TMM factor. Normalized log2-CPM expression values were computed with the standard voom-style correction:

log2-CPM_{ij} = log2((counts_{ij} + 0.5) / (effective_lib_size_j + 1) × 10^6)

For visualization of relative dynamics (main-text Figure 2A and the heatmap in Figure 4A), log2-CPM values were additionally standardized per gene using z-score scaling across the 40 samples.

S5 Differential expression analysis

S5.1 Primary analysis — custom Python limma-voom

Stage-wise differential expression was assessed using a custom implementation of limma-voom in Python. A group-means parameterization was used with a single design-matrix coefficient for each of the six stages. The voom mean-variance weighting was computed from the log2-CPM trend using a cubic spline fit to the mean–variance relationship (Law et al., 2014). Empirical Bayes moderation was applied with *trend=True* and *robust=True* to accommodate heteroscedasticity observed across biological groups (particularly the larger variance in Stage 5 HCC samples). The custom implementation was validated against simulated data with known ground truth, achieving 94% true-positive recall and a Pearson correlation of 0.994 between the recovered and simulated logFC values.

Six biologically motivated stage-wise contrasts were tested:

- Early MASLD vs Control
- MASH vs Early MASLD
- Fibrosis vs MASH
- Chronic-NT vs Fibrosis
- HCC vs Chronic-NT
- HCC vs Control (cumulative disease contrast)

In addition, a longitudinal F-test across the five orthogonal contrasts (any-stage difference, 5 d.f.) was performed to identify genes with non-constant expression across disease progression (Table S2c).

Multiple testing correction was performed using the Benjamini–Hochberg FDR across all tested genes within each contrast. Complete DE results for the IL-10 axis genes are reported in Table S2a.

S5.2 Sensitivity analysis — PyDESeq2

As an orthogonal confirmation, differential expression was re-run with PyDESeq2 v0.5 (Muzellec et al., 2023) using rounded integer counts and the same six stage-wise contrasts (Wald test). Concordance with the primary limma-voom results was high, with Pearson correlations of logFC values across all contrasts ranging from r = 0.88 to 0.98 (Table S2b).

https://doi.org/10.3390/xxxxx

S6 Targeted pathway analysis — IL-10 axis

The analysis focused on a mechanistically defined IL-10/STAT3 axis, comprising eleven genes spanning the canonical pathway and two reported downstream effectors:

- Ligand: *Il10*
- Receptors: *Il10ra, Il10rb*
- Transducers: *Jak1, Jak2, Tyk2, Stat3*
- Negative feedback: *Socs3*
- Co-receptor: *Il6st (gp130)*
- IL-10-responsive effectors: *Scd2, Ddit4* (York et al., 2024)

For each IL-10 axis gene and each stage-wise contrast, we report the moderated log2 fold change, standard error, moderated t statistic, P-value, and BH-adjusted FDR (Table S2). Main-text Figure 2 visualizes these quantities as (A) a forest plot of logFC ± 95% CI, (B) a heatmap of contrast-wise logFC with FDR stars, and (C) a ranked bar plot of the longitudinal F-test significance. Main-text Figure 3 shows the absolute stage-wise expression of each IL-10 axis gene as box + swarm plots.

S7 Cell-type signature enrichment

Because bulk liver RNA-seq conflates changes in cell composition with changes in per-cell expression, a signature-based enrichment analysis was performed to assess cell-type-level remodeling. Importantly, this approach is conceptually distinct from cell-proportion deconvolution: signature scores reflect aggregate expression intensity of curated marker genes, not estimated cellular fractions. Four cell-type signatures with complementary roles in the IL-10 axis were assembled:

- Hepatocytes — parenchymal target of IL-10 signaling via Il10rb and STAT3. Markers: *Alb, Ttr, Apoa1, Apob, Cyp3a11, G6pc, Hnf4a, Serpina1a, Apoc1, Ass1, Fgb, Mup3.*

- Macrophages and monocytes — principal IL-10 producers and responders, including Kupffer cells, monocyte-derived macrophages (MoMFs), and TREM2$^+$ lipid-associated macrophages (LAMs). Markers: *Cd68, Adgre1 (F4/80), Csf1r, Lyz2, Ccr2, Clec4f (Kupffer), Vsig4 (Kupffer), Trem2 (LAM), Itgam (CD11b), Cd9, Fcgr1, C1qa*.

- Natural killer (NK) cells — innate lymphoid source of IL-10 in the NK-rich hepatic environment. Markers: *Klrb1c, Nkg7, Ncr1, Klrk1, Klrd1, Prf1, Gzmb, Ccl5, Eomes*.

- Hepatic stellate cells (HSC) and fibrosis effectors — anti-fibrotic target of IL-10 and canonical fibrosis-associated transcripts. Markers: *Acta2* (α-SMA), *Col1a1, Col1a2, Col3a1, Pdgfrb, Timp1, Lox, Dcn, Lrat, Reln, Des, Ecm1*.

Gene signatures were curated as a consensus panel from CellMarker 2.0 (Hu et al., 2023), filtered to include liver-tissue entries and HCC-related samples, and retained only if cited by at least two independent PMIDs within the same cell type (consensus threshold). Literature-canonical markers were added for MASH-specific or IL-10-relevant subsets (e.g., Trem2, Clec4f, Vsig4 to distinguish resident from monocyte-derived macrophages. Each signature was kept "pure" by excluding pan-macrophage genes (Adgre1, Cd68, Csf1r) from subtype-specific signatures to avoid spurious between-signature correlations. Full gene-level provenance, including primary sources, is reported in Table S3.

Gene-symbol normalization to the current MGI nomenclature was applied because CellMarker 2.0 contains mixed HUGO/MGI symbols and antigen names (e.g., "CD11b" → Itgam; "MHCII" → H2-Aa + H2-Ab1 + H2-Eb1; "α-SMA" → Acta2). The normalization table is included in the GitHub repository as symbol_normalizer.py.

For each sample, the score of a given cell-type signature was computed as the median log2-CPM of its detected signature genes, following the scoring rationale of mMCP-counter (Petitprez et al., 2020). For visualization on a common scale, mean z-scores across samples were also computed and reported alongside the median scores (Table S4a).

https://doi.org/10.3390/xxxxx

S8 Statistical analysis of signature scores

Stage-wise differences in signature scores were assessed using one-way ANOVA (parametric) with Kruskal–Wallis as a non-parametric confirmation, followed by pairwise Mann–Whitney U tests between all fifteen stage pairs (Table S4b–c). Significance of the post-hoc tests was controlled by Holm–Bonferroni correction within each signature. Global F-test significance was additionally adjusted for multiple testing using BH-FDR across the four signatures.

To quantify the relationship between cell-type signature dynamics and IL-10 axis expression, Pearson correlations were computed between each signature score and the log2-CPM of each IL-10 axis gene across all 40 samples. BH-FDR correction was applied over the full 4 × 10 correlation matrix (Table S4d). These correlations underlie Panel D of the main-text Figure 4.

S9 Quality-control framework

Several methodological safeguards were implemented to improve interpretability and reproducibility:

- *Strict metadata filtering: only samples meeting clearly defined criteria for age, diet, STZ treatment, sequencing batch, and tissue were retained, excluding pharmacological-intervention and cross-batch samples (§ S2).*
- *TMM normalization and voom variance weighting (§ S4–S5), which control for library-size and mean-variance effects characteristic of RNA-seq count data.*
- *Orthogonal method replication: the primary limma-voom results were replicated with PyDESeq2 (Table S2b), providing a direct sensitivity check of method dependence.*
- *Robust empirical Bayes moderation:* trend and robust settings were applied to prevent undue influence of the more heterogeneous HCC samples on the variance prior.
- *Family-wise post-hoc adjustment:* all pairwise comparisons on signature scores were Holm–Bonferroni-corrected within each signature to control family-wise error.
- *Sequencing depth and gene detection*: library sizes and the number of genes detected ($\log_2$-CPM > 1) per sample were assessed to confirm uniform sequencing depth and consistent detection across the curated cohort (Figure S1, panels A and B).
- *Global architecture inspection:* principal component analysis on the top 2,000 most variable genes and sample-to-sample Euclidean distance clustering (Ward linkage) were used to inspect global transcriptomic architecture and detect outliers (Figure S1).
- *Biological-marker validation:* stage-specific expression of canonical markers (Afp for HCC, Alb for hepatocyte function, Col1a1 for fibrosis) was confirmed and is included in Figure S1.

S10 Nomenclature and terminology

The cell-type signature analysis in § S7 is reported as "cell-type signature enrichment scoring" rather than "deconvolution", which is the standard term for quantitative cell-proportion estimation methods such as CIBERSORT or CIBERSORTx. The present method reports relative enrichment of gene-set scores between stages and is not calibrated to recover absolute cell proportions. This is a deliberate choice reflecting the analytical limits of bulk RNA-seq.

Disease terminology follows the 2023 MASLD/MASH reclassification (Rinella et al., 2023), with "MASLD" used throughout the text instead of the former "NAFLD/NASH" nomenclature. References to historical literature retain the original terminology when required for accurate citation.

S11 Code and data availability

The custom bioinformatic pipeline — encompassing metadata curation, TMM + log2-CPM normalization, custom limma-voom implementation, PyDESeq2 sensitivity analysis, CellMarker 2.0 + literature-canonical signature enrichment, IL-10 axis correlations, and all figure generation scripts — is openly available at:

https://github.com/Hsolleiro/GSE246221_IL10_Analysis

The repository additionally contains a comprehensive R to Python methodological audit, including an rpy2-based comparison with the reference R limma package on the same cohort data, which yielded Pearson r = 1.000 across all six pairwise contrasts (with top 100 gene overlap of 91–100 per contrast), confirming numerical equivalence of the custom Python implementation.

Raw data are available from GEO (accession GSE246221, https://www.ncbi.nlm.nih.gov/geo/query/acc.cgi?acc=GSE246221). All processed intermediate files, differential expression tables, signature scores, and correlation matrices are provided as Supplementary Tables A1–A4 accompanying this manuscript.

Key methodological references

1. Hu C, Li T, Xu Y, et al. CellMarker 2.0: an updated database of manually curated cell markers in human/mouse and web tools based on scRNA-seq data. Nucleic Acids Research, 2023; 51(D1): D870–D876.
2. Petitprez F, Levy S, Sun C-M, et al. The murine Microenvironment Cell Population counter method to estimate abundance of tissue-infiltrating immune and stromal cell populations in murine samples using gene expression. Genome Medicine, 2020; 12: 86.
3. Law CW, Chen Y, Shi W, Smyth GK. voom: precision weights unlock linear model analysis tools for RNA-seq read counts. Genome Biology, 2014; 15: R29.
4. Muzellec B, Teleńczuk M, Cabeli V, Andreux M. PyDESeq2: a Python package for bulk RNA-seq differential expression analysis. Bioinformatics, 2023; 39: btad547.
5. Robinson MD, Oshlack A. A scaling normalization method for differential expression analysis of RNA-seq data. Genome Biology, 2010; 11: R25.
6. Remmerie A, Martens L, Thoné T, et al. Osteopontin expression identifies a subset of recruited macrophages distinct from Kupffer cells in the fatty liver. Immunity, 2020; 53: 641–657.
7. Jeong S-H, Kim H-B, Kim M-C, et al. A streptozotocin + high-fat-diet mouse model for multistage MASLD/MASH-HCC progression. (GSE246221).
8. Rinella ME, Lazarus JV, Ratziu V, et al. A multisociety Delphi consensus statement on new fatty liver disease nomenclature. Hepatology, 2023; 78: 1966–1986.
9. York AG, Skadow MH, Oh J, et al. IL-10 constrains sphingolipid metabolism to limit inflammation. Nature, 2024; 627: 628–635.
