# Supplementary material for: Hepatocarcinogenesis in Metabolic Dysfunction-Associated Steatotic Liver Disease (MASLD): Emerging Roles of Interleukin-10 and Transcriptomic Insights into IL-10 Signaling Rewiring
Source: Biomedicines. 2026 May 12;14(5):1093. doi: 10.3390/biomedicines14051093 (PMC13204704; doi:10.3390/biomedicines14051093)

**Supplementary Figure S1. Quality control of the curated cohort (n=40, GSE246221)**

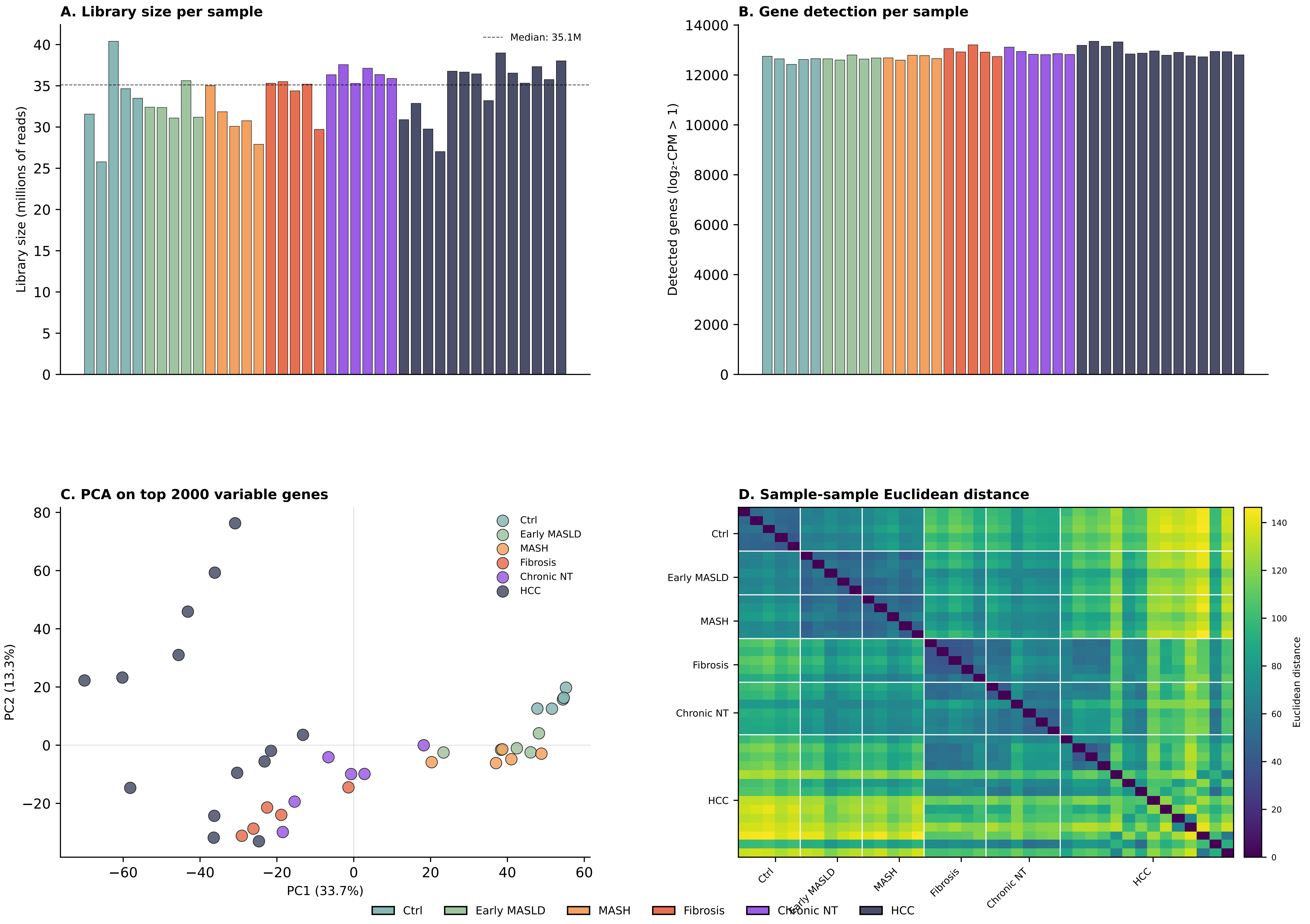

Supplement: Supplementary file 1 [file biomedicines-14-01093-s001.zip › biomedicines-4237763_SuppFigS1.pdf]
